# Editorial: Metabolic crosstalk between cancer cells and immune cells in the tumor microenvironment: cellular and molecular insights, and their therapeutic implications

**DOI:** 10.3389/fonc.2025.1751044

**Published:** 2025-12-11

**Authors:** Parmanand Malvi, Shivendra Vikram Singh, Balkrishna Chaube

**Affiliations:** 1 Department of Biochemistry and Molecular Genetics, University of Alabama at Birmingham, Birmingham, AL, United States; 2 Department of Surgery, St. Jude Children’s Research Hospital, Memphis, TN, United States; 3 Department of Biosciences and Bioengineering, Indian Institute of Technology Dharwad, Karnataka, India

**Keywords:** cancer metabolism, tumor microenvironment, metabolic reprogramming, immune response, immune-cell metabolism, therapeutic target, cancer immunotherapy

## Introduction

Cancer is fundamentally considered as an evolutionary disease characterized by mutation-driven clonal selection, shaped by microenvironmental and therapeutic pressures, leading to heterogeneity, malignant/metastatic progression, and therapy resistance (1–3). Cancer evolution is accompanied by heritable variation (such as point mutations, copy number variation, structural variants, epigenetic changes), selection (e.g., clonal expansion of cells with fitness-enhancing mutations), competition (for example, nutrient/oxygen competition, immune evasion and metabolic competition) and adaptation (such as drug resistance, metastasis and immune escape).

As a part of cancer evolution, metabolic reprogramming is now widely recognized as one of the hallmarks of cancer (4)—not merely as a means by which tumor cells sustain proliferation, but as a central axis by which malignant cells shape their microenvironment and evade immune control (5). Simultaneously, immune cells themselves operate within the metabolic constraints of the tumor microenvironment (TME), adapting—or failing to adapt—to nutrient scarcity, metabolite accumulation, hypoxia, and altered redox states (6). This complex interplay between tumor‐cell and immune‐cell metabolism forms a dynamic and critical interface—one that presents both mechanistic insight and therapeutic opportunity (5, 6).

The Research Topic *“Metabolic Crosstalk between Cancer Cells and Immune Cells in the Tumor Microenvironment: Cellular and Molecular Insights, and their Therapeutic Implications”* encompasses 14 contributions—nine original research papers, four review articles, and one perspective article— collectively illuminating how metabolic pathways in tumor and immune cells converge, compete, and cooperate—and how these interactions could be harnessed in the clinic.

## Articles overview

The contributions to this Research Topic explore how metabolic programs in cancer and immune cells interact in the TME, and how those interactions can be exploited therapeutically. The Research Topic emphasizes lipid, amino-acid and micronutrient metabolism, ferroptosis and redox biology, metabolic biomarkers and gene signatures, and strategies to improve immunotherapy by modulating metabolism. Thus, the Research Topic maps a cross-cutting picture: metabolic programs in tumors are not cell-autonomous — they rewire local and systemic immunity, create metabolic barriers to effective antitumor responses, and offer multiple points for therapeutic intervention (from small-molecule metabolic inhibitors to combination of metabolic and immunotherapy strategies. These works advance our understanding of how metabolism modulates immunity and uncover new therapeutic opportunities for targeting metabolic vulnerabilities.

## Mechanistic insights into the tumor–immune metabolic interface

Cancer cells exploit metabolic plasticity to adapt to nutrient-deprived environments and sustain proliferation (7). A key theme emerging across the original research articles is that metabolic reprogramming in tumor cells has dual purpose: supporting the malignant phenotype and actively modulating the immune microenvironment. Several studies in this Research Topic dissect these intertwined processes at multiple biological aspects.

A clinical-translational study by Jin et al. explored the prognostic implications of metabolic imaging in immunotherapy response among non-small cell lung cancer (NSCLC) patients, revealing that elevated total-body PET/CT metabolic activity correlates with high PD-L1 expression and improved outcomes following checkpoint blockade. This clinical link between tumor metabolic phenotype and immune responsiveness highlights the translational potential of metabolic biomarkers. Further mechanistic data explore tumor‐intrinsic regulators of metabolism and immune milieu. A study by Dal Maso et al. investigated Liver kinase B1 (LKB1), a master regulator of energy homeostasis, demonstrating its association with favorable prognosis and a remodeled immune microenvironment in small cell lung cancer (SCLC). By linking key metabolic regulators to immune infiltration, this work emphasizes the integrated nature of metabolic and immunological regulation in malignancies. These findings highlight how metabolic gatekeepers may modulate the tumor’s immunological landscape.

Mitochondrial metabolism also plays a critical role in cancer (8). A complementary bioinformatics and experimental study by Huang et al. identified mitochondrial metabolism–related genes in cervical cancer that correlate with patient survival and immune infiltration. By integrating transcriptomic and cellular analyses, the authors showcased the mitochondrial–immune axis as a determinant of tumor progression and therapeutic sensitivity. Lipid metabolism is yet another dimension. Liu et al. provided another layer of insight through their integrative analysis of fatty acid metabolic subtypes in gastric cancer, revealing distinct immunometabolic profiles and therapeutic vulnerabilities in these subtypes. Their findings suggest that lipid metabolism may serve as both a biomarker and a therapeutic target in tailoring immunotherapy strategies.

Circulating levels of glucose has been implicated as a major contributor to the occurrence and advancement of malignant tumors. Over a 13-year prospective follow-up study by Li et al., women with higher fasting plasma glucose levels exhibited an increased risk of breast cancer; the study suggests early glucose-control interventions might reduce breast cancer risk. Xu et al. examined ferroptosis—a regulated cell death pathway dependent on lipid peroxidation—within the hypoxic gastric cancer microenvironment. They demonstrated how ferroptosis-related metabolic reprogramming reshapes immune infiltration, offering a framework for combination therapies that exploit ferroptotic sensitivity alongside immune modulation and revealing how regulated cell death mechanisms intersect with immunometabolism.

Using two-sample Mendelian randomization, the study by Li et al. found that genetically predicted levels of various immune cell immunophenotypes causally influence risk of gynecologic malignancies—e.g., B cells panels were protective for cervical cancer, monocyte panels protective for ovarian/vulvar cancer—but reverse causality (cancer → immune cells) was not supported. Adipocytes also play crucial role in various aspects of tumorigenesis (9). The review by Li et al. explores how bone-marrow adipocytes (BMAs) in the bone marrow microenvironment (“soil”) facilitate bone metastasis of lung cancer cells (“seed”), via adipokines, provision of lipids, osteoclast/osteoblast regulation and immune modulation, thus emphasizing the adipokines and metabolic interplay between BMAs and tumor cells.

Collectively, these investigations strengthen the concept that tumor and immune cell metabolisms are not separate trajectories but entwined circuits. Tumor cells not only compete for nutrients (such as glucose, amino acids, lipids) but release signaling intermediates/metabolites (e.g., lactate) that actively suppress or reprogram immune effectors. Conversely, immune cell metabolic fitness—and its underlying mitochondrial, lipid and amino-acid metabolism—determines whether immune responses succeed or falter in the TME.

## Immunometabolic circuits: translational and therapeutic opportunities

The accompanying articles in this Research Topic also underscore the translational potential of mechanistic insights in oncology. By delineating the molecular and metabolic pathways that drive tumor progression and modulate immune responses, these studies pinpoint actionable vulnerabilities within cancer cells and the tumor microenvironment. Such insights provide a foundation for the rational development of targeted therapies and combination strategies, bridging fundamental biology and clinical implications.

Elevated lactate levels within the TME play a critical role in modulating immune cell function in cancer (10). A preclinical study by Gurel et al. in melanoma showed that combining lactate dehydrogenase inhibitors (LDHIs) with immune checkpoint inhibitors (ICIs) enhanced anti-tumor immune responses and displayed greater efficacy in slowing down the tumor growth, suggesting targeting lactate metabolism may boost ICI efficacy. The review article by Sun and Xiao on acetyl‐CoA acetyltransferase 1 (ACAT1) in cancer describes how ACAT1 contributes to tumor initiation, progression, suppression of anti-tumor immunity and proposes ACAT1 inhibition as a therapeutic strategy via targeting cholesterol esterification and tumor immunity. A review on ovarian cancer by Xia et al. discusses how dysregulation of the arachidonic acid metabolic pathways and their metabolites (COX, LOX and CYP450) contributes to tumor initiation, progression, immune-microenvironment alteration, and may serve as a biomarker or therapeutic target.

Aberrant trace-metal metabolism is increasingly recognized as one of the key features of cancer development and progression, influencing proliferation, oxidative stress, immune regulation and therapeutic response (11). A further review by Yao et al. addressed zinc and trace-metal metabolism, an area often overlooked in onco‐immunometabolism. Zinc, a critical cofactor for antioxidant and transcriptional processes, exerts context-dependent effects—supporting immune competence at physiological levels but fostering tumor growth when dysregulated. The authors highlight that targeting zinc metalloproteins and modulating zinc transporter function may open novel avenues for cancer therapy. As a key trace element, copper also plays a vital role in mitochondrial energy production, redox homeostasis, and modulation of programmed cell death. A bioinformatic study by Chen et al. developed a five-gene risk signature (TFRC, SORD, SLC11A2, FKBP4 and AANAT) based on copper metabolism-related genes (CMRGs) in Ewing’s sarcoma; high-risk patients had significantly worse survival, and the signature also correlated with immune cell infiltration and suggested potential drug sensitivities.

Finally, the perspective article by Kang et al. revisited the kynurenine pathway of tryptophan catabolism—a classic mechanism of tumor‐induced immune suppression. The authors propose that dual inhibition of kynurenine pathway’s rate-limiting enzymes indoleamine-2,3-dioxygenase 1 (IDO1) and tryptophan 2,3-dioxygenase-2 (TDO2) as well as downstream metabolites could disrupt the metabolic crosstalk between tumor and immune cells. These therapeutic strategies could better reverse immunosuppression in the TME to overcome drug resistance and to improve therapeutic outcomes. Together, these conceptual frameworks point toward a unifying principle: restoring metabolic balance within the TME is central to effective cancer immunotherapy. They emphasize that successful approaches will require careful temporal and spatial tuning of metabolic interventions, as indiscriminate suppression of metabolism may inadvertently impair immune effectors. By understanding how specific pathways influence immune fate decisions, researchers can design interventions that reprogram the TME toward anti-tumor immunity.

## Challenges and emerging directions

While the collective findings in this Research Topic provide a powerful snapshot of current progress, they also reveal critical gaps and future challenges. First, the metabolic heterogeneity of tumors remains a major barrier. Spatial and temporal variations in nutrient availability, oxygen tension, and metabolite gradients create complex immunometabolic niches. Advances in single-cell multi-omics and spatial metabolomics will be key to dissecting these interactions *in situ*. Second, metabolic targeting must achieve selectivity to avoid systemic toxicity. Many metabolic enzymes are shared between tumor and immune cells, and indiscriminate inhibition may blunt antitumor immunity. Precision approaches that exploit unique dependencies—such as tumor-specific isoforms, differential transporter expression, or context-driven metabolic states—represent promising frontiers. Third, integrating metabolic interventions with established therapies, including immune checkpoint inhibitors, radiotherapy, and targeted drugs, requires nuanced understanding of timing and sequence. As highlighted by Jin et al., Xu et al., and Gurel et al., metabolic states and metabolites levels can predict and even modulate response to immunotherapy. Prospective trials incorporating metabolic imaging and biomarker-guided treatment are warranted. Finally, the field must expand its translational scope. While many studies in this Research Topic are mechanistic or correlative, translating these insights into robust clinical realities remains challenging. Collaborative efforts that integrate metabolic imaging, metabolite profiling, immune phenotyping, clinical oncology, and computational biology will accelerate the translation of immunometabolic discoveries into therapeutic advances. This integrated approach may allow stratification of patients likely to benefit from metabolic–immunotherapy combinations.

## Concluding remarks

The contributions assembled in this Research Topic collectively feature the importance of metabolism as a language of communication within the tumor microenvironment. From amino acids and lipids to lactate and kynurenine, these metabolites shape the immune landscape, influencing whether tumors thrive or regress.

As the boundaries between cancer cell metabolism and immune regulation blur, it becomes increasingly clear that therapeutic success will depend on decoding—and ultimately rewriting—this metabolic dialogue. The studies presented here provide not only mechanistic insights but also translational pathways to exploit metabolic vulnerabilities for clinical gain.

In summary, this Research Topic reflects the vibrant and rapidly evolving field of cancer immunometabolism. It is our hope that this Research Topic will stimulate deeper cross-disciplinary collaboration among metabolism researchers, immunologists and clinicians. It reaffirms that metabolic crosstalk is not merely a consequence of tumor growth but a driving force of tumor–immune dynamics. Continued integration of metabolic research with immunotherapy promises to unlock new frontiers in precision oncology.
